# Developing a European framework for the prevention of communicable diseases: three points for attention

**DOI:** 10.2807/1560-7917.ES.2024.29.43.2400306

**Published:** 2024-10-24

**Authors:** Daniel H de Vries, Mandy Geise, Anna Christina Maukner, Piotr Kramarz, Charlotte Deogan, John Kinsman

**Affiliations:** 1 Amsterdam Institute for Global Health and Development, Amsterdam, the Netherlands; 2 International Institute of Social Studies, Erasmus University Rotterdam, Rotterdam, the Netherlands; 3 European Centre for Disease Prevention and Control (ECDC), Stockholm, Sweden

**Keywords:** prevention, communicable diseases, behavioural sciences, social sciences, ECDC, framework

## Abstract

The capacity to deliver programmes that prevent and control infectious diseases is a key public health function. The European Centre for Disease Prevention and Control (ECDC) aims to support and strengthen this capacity in European Union/ European Economic Area (EU/EEA) countries as part of its 2021–27 strategy which includes explicit attention to social and behavioural aspects of disease prevention. To achieve its strategic goals, it is important that ECDC improves its knowledge of prevention strategies, actors and activities in EU/EEA countries. In this Perspective, we summarise three challenges to implementing the prevention framework proposed by ECDC: (i) defining, recognising and identifying with ‘prevention’, (ii) integrating new understandings into established ways of thinking, and (iii) the need for more attention to prevention in governance. These challenges are derived from the findings of a project which conducted a preliminary mapping of prevention actors, networks and activities in four EU countries to support the development of a community of practice within the new ECDC prevention framework. This Perspective serves to draw attention to this prevention framework and the three identified challenges for those working on its implementation.

## Background

The European Centre for Disease Prevention and Control (ECDC)’s amended mandate, adopted by the European Parliament on 4 October 2022 and by the European Council on 24 October 2022, provides a renewed commitment to the area of communicable disease prevention and control [1,2]. The amended mandate, in addition to other concerns, calls on ECDC to develop a ‘framework for the prevention of communicable diseases and related special issues, including socio-economic risk factors, vaccine preventable diseases, antimicrobial resistance, health promotion, health education, health literacy and behaviour change’ [3]. ECDC proposes a working definition of ‘prevention’ of communicable diseases as specific interventions that aim to avoid the initiation of outbreaks or limit increases in disease incidence or consequences and thereby minimise the burden of communicable diseases. The objectives of this Perspective are to draw attention to this new ECDC prevention framework and to highlight three identified challenges for those working on its implementation.

A range of different prevention categories exists, from primordial to quaternary forms of prevention [3-6] (Table). The five categories are not mutually exclusive, and crossover may be seen in some areas. For example, antimicrobial resistance (AMR) and prescribing behaviour could be seen as having an impact in both primordial and quaternary prevention. Similarly, ‘treatment as prevention’ with tuberculosis (TB) can be seen as both a tertiary and primary (or possibly primordial) prevention action. Combined, these five prevention categories aim to prevent the onset of disease through general risk reduction, the creation of healthy environments and address the downstream sequelae of a manifested disease.

**Table:** Categories of prevention

Category	Definition
**Primordial**	Reducing risk factors across a whole population while focusing on social and environmental factors. The term is usually applied to addressing common risk factors and upstream determinants of infectious diseases, such as climate, environmental, social-behavioural, and healthcare systems. For example, changes to laws and national policies, or behavioural or social change strategies to reduce poverty or antibiotic usage, promote treatment as prevention, or foster policies for healthy lifestyles (a core pillar of health promotion).
**Primary**	Steps taken to protect a vulnerable community or person by avoiding occurrence of disease. This can be achieved through, for example, immunisation against disease or measures such as pre-exposure prophylaxis (PrEP) for HIV or behavioural change interventions to reduce vaccine hesitancy. Health and risk communication may also be considered primary prevention.
**Secondary**	Methods or approaches to increase detection and address an existing disease before the appearance of symptoms, in order to avoid the beginning of illness. Examples include screening for latent TB infection, HIV, or genital chlamydia infection. A social-behavioural example may be stigma-reduction measures which motivate people to visit screening sites.
**Tertiary**	Reducing the severity of symptomatic disease conditions and any accompanying complications through rehabilitation and treatment. Physical therapies that support the rehabilitation of children with paralytic polio, treatment for prevention, or psychological interventions against depression or anxiety among patients with long COVID are examples of tertiary prevention.
**Quaternary**	Methods to mitigate or avoid results of unnecessary or excessive interventions within the health system. These include actions taken to identify a patient at risk of overmedicalisation in order to protect the patient from new medical invasion, and to suggest interventions which are ethically acceptable. Antimicrobial stewardship, including behavioural change approaches to antibiotic prescription, may be seen as examples of quaternary prevention.

Adapted from [3,14].

## Mapping the prevention architecture

ECDC’s extended mandate specifically identifies primary prevention for vaccine preventable diseases and quaternary prevention for AMR as the priority disease and related special issues areas for the prevention framework, with an additional aim to address both these areas through primordial prevention activities such as health education. This reflects a paradigm shift in a western medical context where treatment is often seen as prioritised over prevention [7]. This broader public health approach to communicable disease prevention acknowledges the importance of considering structural factors that can affect the vulnerability of people to communicable diseases, and as such it also links disease prevention to social change [8,9]. It also underlines a key role of social and behavioural sciences as a basis for enhancing public understanding of and adherence to preventive measures, complementing more traditional health sciences and facilitating a move towards a wider discourse and range of actions.

Implementing this framework will require increased collaboration and knowledge exchange among prevention actors across public health authorities, universities and research institutions, civil society and community-based organisations. In order to identify these actors, ECDC initiated a short project to pilot a methodology to map relevant stakeholders and obtain basic information about the prevention architecture in European Union (EU) countries. The pilot project was conducted in four countries (Austria, Cyprus, the Netherlands and Poland) from October 2022 to March 2023 using a combination of in-depth qualitative interviews (n = 16) with actors from the government, academia and non-government organisations (NGOs), desk research and an online survey (n = 200). Interviews were analysed using qualitative software (Dedoose version 9.2.014, Los Angeles, United States) and the basic survey results using Microsoft Excel. Participants were recruited either through desk review and snowball sampling (pilot 1, Austria and Cyprus) or through an online self-registration portal with an outreach campaign (pilot 2, the Netherlands and Poland). While not a rigorous study, the results of this exercise not only tested several mapping methodologies, but also illuminated three challenges which we believe are important for further work on disease prevention in the EU/European Economic Area (EEA). It is the latter which we highlight in the remainder of this perspective.

## Three challenges to a wider prevention agenda

### Challenge 1: defining, recognising and identifying with ‘prevention’

While most respondents in our survey self-classified as working in primary or secondary prevention (Figure), throughout the project it became clear that many actors in the field struggled to define themselves as working in prevention at all. During the interviews, some participants replied that they were not sure they were in fact the right person to talk to, as they themselves did not see their work as falling into this category. For example, a professional replied that while their department is called Health Promotion and Disease Prevention, their focus had mainly been on health promotion, “and as such there are no activities specifically regarding infectious disease prevention.”

**Figure:**
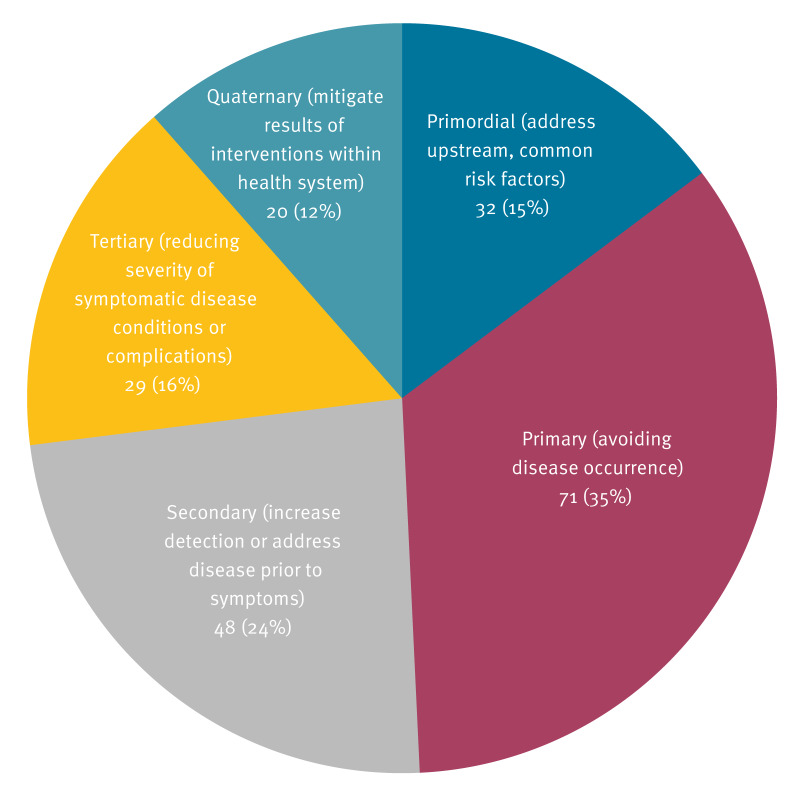
Percentage of survey participants self-classifying relative to different levels of prevention, Austria, Cyprus, the Netherlands and Poland, October 2022–March 2023 (n = 200)

Another example of the challenge in self-identification as a prevention actor was given by a representative from an NGO in one of the participating countries which provided support for medical care to refugees and migrants, but who still declined to participate in the interview on the basis that they were not involved in the field of communicable disease prevention. This was despite the observation by the researchers that the NGO representative’s activities included the provision of information about COVID-19 and vaccinations, accompanying people to hospitals, acting as translator/mediator, giving assistance to new mothers, providing essential medicines and medical services and conducting cultural mediation in hospitals.

This difficulty of defining prevention also extended to the desk reviews where the research team struggled to determine the difference between ‘prevention’ and ‘control’ at times. Often, overlap between these concepts was found. Some activities, such as surveillance and monitoring, seemed at first glance to clearly fall into control, yet at the same time could be considered part of secondary prevention activities. Some participants also mentioned treatment, which could be seen as tertiary prevention. Prevention actors also often reported that they were working in both prevention and control departments, and as such the distinction between these fields at times appeared artificial. Even though prevention and control are two domains with different rationales and conceptualisations, activities may sometimes fall within both these domains. Actors would say they were not working on prevention but on control, and immediately name activities they were doing which fell under ‘prevention’ within ECDC's definition.

### Challenge 2: integrating new understandings into established ways of thinking

A key issue the research team encountered was that while there is increasing attention given to social and behavioural sciences in prevention and control, the importance of these disciplines is not fully established and not consistently integrated into prevention and control efforts. This is despite decades of behavioural and social science research identifying factors that can inhibit or facilitate communicable disease prevention, and the availability of many tools, methods, concepts and approaches [10-12].

Reasons for this may be that the traditional understanding of prevention in public health has been strongly informed by biomedical and epidemiological research, and that knowledge generated in this area has been siloed in disciplines [13]. As a result, biomedical and epidemiological knowledge-gathering tends to provide the bulk of the evidence for implementing health programming. For example, in one country, several participants described how a key prevention challenge was the lack of a genomic surveillance system to track diseases: “there is a huge gap in biomonitoring of zoonotic and parasitic diseases.”

It does not come as a surprise then that a range of social and behavioural science training needs was identified by respondents, including topics such as stigma and discrimination, communication, interdisciplinary collaboration in crisis management, preparedness for communicable disease outbreaks, building leadership and trust, community engagement, enhancing public awareness, building health literacy and capacity or promoting access to care for key populations.

### Challenge 3: the need for more attention to prevention in governance

The commonly expressed need among participants was for stronger national coordination and governance in the field of prevention. Challenges mentioned included the need for stronger collaboration between the national and local public health authorities, as well as with academia and non-governmental institutions, and a need for better funded, independent public health institutes to “make a real change in communicable disease prevention”. Participants highlighted the importance of a shift to a more multidisciplinary approach that explicitly includes social and behavioural sciences and that focuses on structural and societal factors that can impact prevention: “there is a need to bring together all relevant areas of expertise… [to facilitate] better cooperation between the entire chain of care and public health”. A call was also made for more transparency on how decisions are made and how policies on prevention are created, especially given the different COVID-19 prevention measures in the different EU countries.

Participants noted that in some EU countries, the abundance of decentralised prevention activities would benefit from coordination and alignment between different actors to avoid unnecessary overlap or competition. An additional challenge expressed for less-resourced EU countries and non-profit organisations working in the field of prevention was the externally imposed requirements that come with project grants which make it difficult to maintain independence, act speedily according to needs identified on the ground or ensure sustainable activities. Ongoing state-level operational funding was seen as crucial to secure operations for projects and services. It was also expressed that actors working on prevention from a social or behavioural science perspective are often disproportionately affected by the impacts of funding constraints.

## Conclusion

The new strategic ECDC framework for the prevention of communicable diseases, including social and behavioural perspectives, has the potential to provide support for EU countries and comprehensively strengthen prevention activities, implementing lessons learnt from the COVID-19 pandemic. Yet, as we observed during this work, developing a platform for collaboration and coordination on prevention will require addressing some complex challenges. These start with the need to collectively reflect on the multiplicity of meanings and practices that encompass the field of ‘prevention’. Clarity of definition alone may not be enough here. In a context where integration of social and behavioural sciences has become increasingly accepted, transdisciplinary dialogues may uncover competing conceptualisations of what ‘prevention’ may mean in different contexts. In addition, issues of power need to be brought to the centre to address the instances of unequal distribution of attention to treatment over prevention, and to the biomedical over social and behavioural sciences, within some healthcare and public health systems. Clearly, these types of shifts are also relevant for the reassessment of budgets and funding. Through the Community of Practice on Prevention that ECDC will launch in November 2024 (https://prevention.ecdc.europa.eu/), it is hoped that the new ECDC framework for prevention will provide systematic guidance and support to EU countries in their further development, implementation, monitoring and evaluation of strategies and activities related to communicable disease prevention.
